# Case of Suffocation

**Published:** 1853-09

**Authors:** G. R. B. Horner

**Affiliations:** Surgeon U. S. N.


*Case of Suffocation.* Reported by G. R. B. Horner, M. D., Surgeon U. S. N.

Sunday, June 10th, in the morning, Mary Berry, a girl about seven years old, residing in the south-eastern part of Philadelphia, put the top of a broken glass decanter-stopper into her mouth. The top was globular, and about two inches around. From some unknown cause she took a long inspiration, and, as she says, sucked it down her throat. It lodged at the entrance of the œsophagus, and so obstructed that or the glottis, that she immediately became strangled. Her mother and others about her in vain endeavored to relieve her; an apothecary was sent for, and could not do so. Luckily, Mr. Isaac Hugg, an ingenious, long, slender-fingered tailor, living opposite, in Second street, heard the alarm, ran to the poor child's relief, and understanding what had happened, thrust his fingers into her throat, but at first could not feel the stopper. He tried a second time: after raising her feet upwards, her head downwards, and over his knees, and after getting a finger under a projecting point of the broken surface of the stopper, succeeded in throwing it upon the floor. By this time the child was insensible, but on the introduction of his fingers, gagged, assisted his efforts, and was resuscitated, though pronounced dead by the druggist, deceived perhaps by the lividness of her face, and other fatal signs. Of the above facts I was informed while passing her residence at the time of the accident, and by subsequent enquiry.

